# Early detection of gastric cancer after *Helicobacter pylori* eradication due to endoscopic surveillance

**DOI:** 10.1111/hel.12503

**Published:** 2018-06-20

**Authors:** Kosuke Sakitani, Toshihiro Nishizawa, Masahide Arita, Shuntaro Yoshida, Yosuke Kataoka, Daisuke Ohki, Hiroharu Yamashita, Yoshihiro Isomura, Akira Toyoshima, Hidenobu Watanabe, Toshiro Iizuka, Yutaka Saito, Junko Fujisaki, Naohisa Yahagi, Kazuhiko Koike, Osamu Toyoshima

**Affiliations:** ^1^ Department of Gastroenterology Toyoshima Endoscopy Clinic Tokyo Japan; ^2^ The Institute for Adult Diseases Asahi Life Foundation Tokyo Japan; ^3^ Department of Gastroenterology Tokyo Medical Center National Hospital Organization Tokyo Japan; ^4^ Department of Gastroenterology Graduate School of Medicine The University of Tokyo Tokyo Japan; ^5^ Department of Gastrointestinal Surgery Graduate School of Medicine The University of Tokyo Tokyo Japan; ^6^ Department of Gastroenterology Kanto Central Hospital Tokyo Japan; ^7^ Department of Colorectal Surgery Japanese Red Cross Medical Center Tokyo Japan; ^8^ Department of Pathology PCL Japan Tokyo Japan; ^9^ Department of Gastroenterology Toranomon Hospital Tokyo Japan; ^10^ Endoscopy Division National Cancer Center Hospital Tokyo Japan; ^11^ Department of Gastroenterology Cancer Institute Hospital Tokyo Japan; ^12^ Division of Research and Development for Minimally Invasive Treatment Cancer Center Keio University School of Medicine Tokyo Japan

**Keywords:** endoscopic surveillance, gastric cancer, *H. pylori* eradication, mortality rate

## Abstract

**Background:**

*Helicobacter pylori* eradication therapy is commonly performed to reduce the incidence of gastric cancer. However, gastric cancer is occasionally discovered even after successful eradication therapy. Therefore, we examined the prognosis of gastric cancer patients, diagnosed after successful *H. pylori* eradication therapy.

**Materials and Methods:**

All‐cause death rates and gastric cancer‐specific death rates in gastric cancer patients who received successful *H. pylori* eradication treatment was tracked and compared to rates in patients who did not receive successful eradication therapy.

**Results:**

In total, 160 gastric cancer patients were followed‐up for up to 11.7 years (mean 3.5 years). Among them, 53 gastric cancer patients received successful *H. pylori* eradication therapy prior to gastric cancer diagnosis. During the follow‐up period, 11 all‐cause deaths occurred. In the successful eradication group, the proportion of patients with cancer stage I was higher. The proportions of patients who received curative endoscopic therapy and endoscopic examination in the 2 years prior to gastric cancer diagnosis were also higher in the successful eradication group. Kaplan–Meier analysis of all‐cause death and gastric cancer‐specific death revealed a lower death rate in patients in the successful eradication group (*P* = .0139, and *P* = .0396, respectively, log‐rank test). The multivariate analysis showed that endoscopy within 2 years before cancer diagnosis is associated with stage I cancer.

**Conclusions:**

Possible early discovery of gastric cancer after *H. pylori* eradication due to regular endoscopic surveillance may contribute to better prognosis of patients with gastric cancer.

## 1 INTRODUCTION

*Helicobacter pylori* infection is one of the most common triggers of gastric cancer, and gastric cancer is the major cause of cancer deaths.^1^, ^2^, ^3^, ^4^ Gastric cancers are the third leading cause of cancer mortality and advanced disease carries a dismal prognosis with a 5‐year overall survival rate of less than 5%.^5^, ^6^ Surgery and endoscopic resection at an early stage is still the only chance for cure.^7^, ^8^ Although it has been reported that *H. pylori* eradication therapy reduces the incidence of gastric cancer and is widely conducted to prevent gastric carcinogenesis, gastric cancers are still diagnosed in patients who received successful eradication therapy.^9^, ^10^, ^11^, ^12^, ^13^ Thus, endoscopic surveillance of gastric cancers after *H. pylori* eradication is expected to be a beneficial approach for detection. The endoscopic and histological features of gastric cancers after eradication have been investigated vigorously.^14^, ^15^, ^16^ To the best of our knowledge, research on gastric cancer deaths after successful *H. pylori* eradication therapy is insufficient. Therefore, we examined the prognosis of gastric cancer diagnosed after successful *H. pylori* eradication therapy.

## 2 METHODS

### 2.1 Patients

Gastric cancer patients diagnosed at Toyoshima Endoscopy Clinic were analysed retrospectively using an endoscopic database and clinical charts. Esophagogastroduodenoscopy was performed by certificated endoscopists. The patients underwent esophagogastroduodenoscopy either for screening, for a previous history of esophagogastroduodenal disease, present symptoms or abnormal findings on barium meal examination. Biopsy specimens were taken from lesions suspected to be gastric cancer, and the final diagnosis of gastric cancer was based on pathology results. Gastric cancers were classified pathologically as either intestinal or diffuse type according to Lauren's classification.^17^ Data concerning 181 consecutive gastric cancer patients diagnosed between July 2005 and April 2017 were considered for analysis. Because the Toyoshima Endoscopic Clinic is an outpatient clinic, gastric cancer patients are admitted to hospitals such as the National Cancer Center, university hospitals, or core hospitals in areas considered optimal for treatment. The pathological results of surgical specimens and final outpatient information on patients treated for gastric cancer from these hospitals, as recorded in medical charts in the Toyoshima Endoscopy Clinic, were used for this study. Follow‐up of the cohort ended in August 2017. The lost to follow‐up date was defined as the final visit. Patients with the following criteria were excluded: (1) gastric cancer occurring postoperatively in the stomach (n = 6), (2) esophagogastric junction carcinoma (n = 10), (3) patients with unknown method of treatment for gastric cancer (n = 4), and (4) patients with unknown *H. pylori* status (n = 1). Ultimately, 160 gastric cancer patients were analysed. This retrospective study was approved by the Ethical Review Committee of Hattori Clinic on September 7, 2017. Written informed consent was obtained from all patients. All clinical investigations were conducted according to the ethical guidelines of the Declaration of Helsinki.

### 2.2 Clinicopathological assessment

Clinicopathological findings, including the interval since *H. pylori* eradication, interval since last endoscopy, age, sex, body mass index (BMI), past history of cancer (other than gastric cancer), first degree family history of gastric cancer,^12^ history of smoking [Brinkman index = daily amount of tobacco (pieces/d) × period of smoking (years)] and current alcohol intake (g/d), mucosal atrophy [according to Kimura‐Takemoto classification; we divided gastric mucosal atrophy into 6 grades (C‐I, C‐II, C‐III, O‐I, O‐II, and O‐III) based on endoscopic findings],^18^ tumor size, the Union for International Cancer Control cancer stage, location and histological type, were reviewed. The interval since last endoscopy was defined as the interval between the endoscopy during which the gastric cancer was detected and the previous endoscopy.

### 2.3 *H. pylori* infection status

*Helicobacter pylori* infection was confirmed when any one of the following tests was positive; ^13^C‐urea breathe test, stool antigen analysis or *H. pylori* ‐specific immunoglobulin G antibodies in the serum. Patients in whom *H. pylori* infection was confirmed underwent eradication therapy. Patients in whom eradication therapy had failed received additional treatment: the first‐line regimen comprised a proton pump inhibitor (PPI), amoxicillin, and clarithromycin; the second‐line regimen comprised a PPI, amoxicillin, and metronidazole. At least 4 weeks after the completion of eradication therapy, cure status was confirmed via a ^13^C‐urea breath test. Patients were divided into 4 groups according to their *H. pylori* status, history of *H. pylori* eradication therapy, and gastric mucosal atrophy at the time of gastric cancer diagnosis. These groups were: (1) persistent infection group (*H. pylori* infection positive patients, including eradication therapy failure patients), (2) successful eradication therapy group (no *H. pylori* infection patients with *H. pylori* eradication therapy history), (3) spontaneous eradication group (no *H. pylori* infection patients without history of eradication therapy with atrophic gastritis) and (4) uninfected group (no *H. pylori* infection patients without eradication therapy history and gastric atrophy).^19^ We removed groups (3) and (4) from the analysis comparing the effect of *H. pylori* eradication.

### 2.4 Statistical analysis

All statistical analyses were performed using JMP10 software (SAS Institute, Cary, NC, USA). Welch's *t* test was used to compare the means of continuous variables. Comparisons of nominal variables were performed using the χ^2^ test or Fisher's exact test, as appropriate. The incidence of all‐cause death was evaluated using the Kaplan–Meier method, and the statistical significance of the differences was evaluated by log‐rank test. All‐cause death was the primary endpoint, and data were censored at the final visit. Additional endpoint was gastric cancer death. Odds ratios (OR) with 95% confidence intervals (CI) were used as a measure of association and were adjusted using unconditional logistic regression models. A two‐sided *P* ‐value of <.05 was considered to indicate statistical significance.

## 3 RESULTS

### 3.1 Baseline characteristics of 160 gastric cancer patients

The baseline characteristics of the gastric cancer patients are provided in Table 1. In total, 160 gastric cancer patients [90 males and 70 females, mean age 64.6 years (range 30‐89), and mean BMI 22.5 kg/m^2^] were analyzed. All the patients were in good physical condition and could walk independently and visit our clinic. Among them, 59 patients (36.9%) underwent endoscopy examination within the 2 years prior to gastric cancer diagnosis. At the time of gastric cancer diagnosis, 89 patients (55.6%) formed a persistent *H. pylori* infection group, 53 patients (33.1%) formed a successful *H. pylori* eradication therapy group, 12 patients (7.50%) formed a spontaneous eradication group, and 6 patients formed an uninfected group. Patients were followed‐up for up to 11.7 years (mean 3.5 years). Of all the 160 gastric cancer patients, 135 patients (84.4%) were graded as cancer stage I, and 88 patients (55.0%) received curative endoscopic treatment.

**Table 1:** Characteristics of the 160 gastric cancer patients

Characteristics	Total (n = 160)	Death (n = 11)	Survival (n = 149)	*P*
Mean age (range), y	64.6 (30‐89)	70.7 (43‐87)	64.1 (30‐89)	.1584
Sex, n (%)				
Female	70 (43.8%)	7 (63.6%)	63 (42.3%)	.2134
Male	90 (56.3%)	4 (36.4%)	86 (57.7%)	
Mean BMI (range), kg/m^2^	22.5 (15.9‐32.8)	22.3 (15.9‐31.6)	22.6 (16.7‐32.8)	.8400
Past history of cancer, yes	28 (17.5%)	1 (9.09%)	27 (18.1%)	.6907
Family history of gastric cancer, yes	47 (29.4%)	3 (27.3%)	44 (29.5%)	.7030
Smoking				
None	105 (65.6%)	7 (63.6%)	98 (65.8%)	.8900
Brinkman Index <400	21 (13.1%)	2 (18.2%)	19 (12.8%)	
Brinkman Index ≥400	29 (18.1%)	2 (18.2%)	27 (18.1%)	
Alcohol				
None	63 (39.4%)	6 (54.5%)	58 (38.9%)	.6259
<20 g/d	46 (28.8%)	2 (18.2%)	44 (29.5%)	
≥20 g/d	45 (28.1%)	3 (27.3%)	42 (28.2%)	
Endoscopy within 2 y, yes	59 (36.9%)	0	59(39.6%)	.00738^a^
*Helicobacter pylori* status				
Persistent infection	89 (55.6%)	10 (90.9%)	79 (53.0%)	.0400^a^
Successful eradication therapy	53 (33.1%)	0	53 (35.5%)	
Eradicated spontaneously	12 (7.50%)	1 (9.09%)	11 (7.38%)	
Uninfected	6 (3.75%)	0	6 (4.03%)	
Atrophic gastritis				
None	6 (3.75%)	0	6 (4.03%)	.5967
C‐1	0	0	0	
C‐2	16 (1.00%)	0	16 (10.7%)	
C‐3	9 (5.63%)	0	9 (6.04%)	
O‐1	18 (11.9%)	0	18 (12.1%)	
O‐2	27 (16.9%)	3 (27.9%)	24 (16.1%)	
O‐3	84 (69.3%)	8 (72.7%)	76 (51.0%)	
Stage, n (%)				
I	135 (84.4%)	3 (27.3%)	132 (88.6%)	<.0001^a^
II	11 (6.88%)	0	11 (7.38%)	
III	7 (4.38%)	2 (18.2%)	5 (3.36%)	
IV	7 (4.38%)	6 (54.5%)	1 (0.67%)	
Location				
Upper third	30 (18.8%)	6 (54.5%)	24 (16.1%)	.0034^a^
Middle third	71 (44.4%)	1 (9.09%)	70 (47.0%)	
Lower third	59 (36.9%)	4 (36.4%)	55 (36.9%)	
Mean size (range), mm	28.9 (1.0‐160)	68.1 (10‐120)	26.1 (1.0‐160)	.0061^a^
Pathology				
Intestinal type	114 (71.3%)	3 (27.9%)	111 (74.5%)	.0022^a^
Diffuse type	46 (28.8%)	8 (72.7%)	38 (25.5%)	
Treatment for gastric cancer				
Endoscopy	88 (55.0%)	2 (18.2%)	86 (57.7%)	<.0001^a^
Surgery	61 (38.1%)	1 (9.09%)	60 (40.3%)	
Chemotherapy	5 (3.13%)	4 (36.4%)	1 (0.67%)	
Best supportive care	2 (1.25%)	2 (18.2%)	0	
Observation	4 (2.50%)	2 (18.2%)	2 (1.34%)	
Observation period (range), y	3.47 (0.08‐11.7)	1.55 (0.15‐3.69)	3.69 (0.08‐11.7)	<.0001^a^

BMI, body mass index.

a Statistically significant.

During the follow‐up period, 11 all‐cause deaths were observed. Among the 11 gastric cancer patients, 7 patients died because of gastric cancer and the remaining 4 patients died from other diagnoses including pneumonia, myocardial infarction, pharyngeal cancer, and senility. Characteristics of the 11 all‐cause deaths are presented in Table S1. Mean age, the proportion of males, mean BMI, past history of cancer, family history of gastric cancer, smoking habit, alcohol intake, and atrophic gastritis were not significantly different in the all‐cause death group (n = 11) and survival group (n = 149). Among the 11 all‐cause deaths, only 27.3% (3/11) were stage I, compared with 88.6% (132/149) in the survival group (*P* < .0001). A greater proportion of lesions were in the upper third region in the all‐cause death group (54.5% vs 16.1% [survival group], *P* = .0034). The same result was seen for diffuse type gastric cancer pathology (72.7% vs 25.5%, *P* = .0022) and mean gastric cancer size (68.1 mm vs 26.1 mm, *P* = .0061). There were no patients with a history of successful *H. pylori* eradication therapy or endoscopy within 2 years prior to gastric cancer diagnosis in the all‐cause death group.

### 3.2 Gastric cancer in the *H. pylori* persistent infection group vs the successful eradication group

Next, we examined the influence of successful *H. pylori* eradication therapy on all‐cause death and gastric cancer‐specific death in gastric cancer patients. We excluded the 6 uninfected gastric cancer patients (who had no *H. pylori* infection or history of eradication therapy and gastric atrophy) from this analysis because this small fraction of gastric cancer patients displayed multi‐fractional carcinogenesis without *H. pylori* infection.^20^ We also excluded the 12 patients in the *H. pylori* spontaneous eradication group (no *H. pylori* infection patients without history of eradication therapy with atrophic gastritis).^21^, ^22^

As shown in Table 2, 53 gastric cancer patients were diagnosed after successful *H. pylori* eradication therapy (the mean interval between the eradication treatment and the detection of gastric cancer was 4.3 years). Mean age, the proportion of males, mean BMI, past history of cancer, family history of gastric cancer, smoking habit, alcohol intake, and atrophic gastritis were not significantly different in the *H. pylori* persistent infection group (n = 89) and successful eradication group (n = 53). In the 89 *H. pylori* persistent infection group, 23.6% (21/89) were stage II or greater compared with only 5.66% (3/53) in the successful *H. pylori* eradication group (*P* = .0251). The rates of diffuse type gastric cancer and the mean gastric cancer sizes were larger in the *H. pylori* persistent infection group compared to the successful *H. pylori* eradication group (*P* = .0022 and *P* < .0001, respectively). The proportion of patients who received curative endoscopic therapy for gastric cancer and screening endoscopy in the 2 years prior to gastric cancer diagnosis was higher in the successful eradication group (*P* = .0022 and *P* < .0001, respectively).

**Table 2:** Characteristics of *Helicobacter pylori* persistent infection and successful eradication group

Total (n = 142)	Infection (n = 89)	Eradication (n = 53)	*P*
Mean age (range), y	64.1 (30‐89)	64.9 (37‐84)	.6942
Sex, n (%)			
Female	40 (44.9%)	21 (63.6%)	.5355
Male	49 (55.1%)	32 (36.4%)	
Mean BMI (range), kg/m^2^	22.3 (17.8‐32.1)	22.7 (15.9‐32.4)	.4060
Past history of cancer, yes	12 (13.5%)	11 (20.8%)	.2553
Family history of gastric cancer, yes	21 (23.6%)	18 (34.0%)	.1806
Smoking			
None	57 (64.0%)	37 (69.8%)	.2560
Brinkman Index <400	8 (8.99%)	8 (15.1%)	
Brinkman Index ≥400	20 (22.5%)	7 (13.2%)	
Alcohol			
None	30 (33.7%)	21 (39.6%)	.6695
<20 g/d	26 (29.2%)	17 (32.1%)	
≥20 g/d	29 (32.6%)	14 (26.4%)	
Endoscopy within 2 y	12 (13.5%)	42 (79.2%)	<.0001^a^
Atrophic gastritis			
C‐1	0	0	.0599
C‐2	5 (5.62%)	9 (17.0%)	
C‐3	4 (4.49%)	4 (7.55%)	
O‐1	8 (8.99%)	9 (17.0%)	
O‐2	18 (20.2%)	8 (15.1%)	
O‐3	54 (60.7%)	23 (43.4%)	
Stage, n (%)			
I	68 (76.4%)	50 (94.3%)	.0251^a^
II	10 (11.2%)	3 (5.66%)	
III	5 (5.62%)	0	
IV	6 (6.74%)	0	
Location			
Upper third	21 (23.6%)	5 (9.43%)	.0761
Middle third	33 (37.1%)	27 (50.9%)	
Lower third	35 (39.3%)	21 (39.6%)	
Mean size (range), mm	38.3 (3.0‐180)	16.9 (1.0‐100)	<.0001^a^
Pathology			
Intestinal type	56 (62.9%)	46 (86.8%)	.0022^a^
Diffuse type	33 (37.1%)	7 (13.2%)	
Treatment for gastric cancer			
Endoscopy	38 (42.7%)	39 (73.6%)	.0022^a^
Surgery	42 (47.2%)	13 (24.5%)	
Chemotherapy	5 (5.62%)	0	
Best supportive care	2 (2.25%)	0	
Observation	2 (2.25%)	1 (1.89%)	

BMI, body mass index.

a Statistically significant.

**Figure 1:**
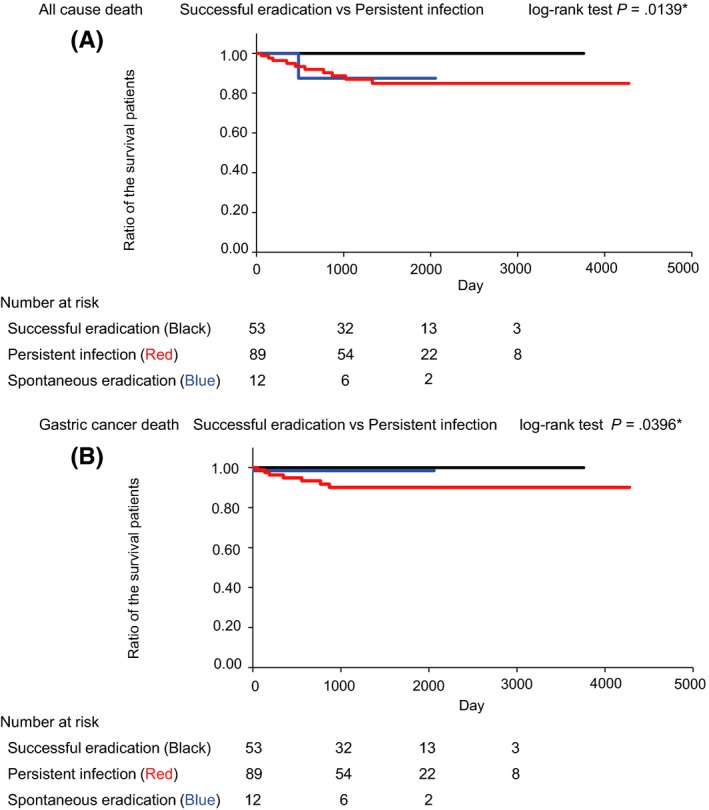
Kaplan–Meier analysis of the proportions of patients free of death in the *Helicobacter pylori* persistent infection group and successful *H. pylori* eradication group. A, Kaplan–Meier analysis and log‐rank test of the proportions of patients free of all‐cause death in the *H. pylori* persistent infection group and the successful *H. pylori* eradication group. B, Kaplan–Meier analysis and log‐rank test of the proportions of patients free of gastric cancer death in the *H. pylori* persistent infection group and the successful *H. pylori* eradication group

Kaplan–Meier analysis of the proportions of patients free of all‐cause death in the *H. pylori* persistent infection group and successful *H. pylori* eradication group is shown in Figure 1A. A significant difference was found for the incidence of all‐cause death in these 2 groups using a log‐rank test (*P* = .0139). Kaplan–Meier analysis of the proportions of patients free of gastric cancer death in the *H. pylori* persistent infection group and successful *H. pylori* eradication group is shown in Figure 1B. A significant between‐group difference was also found using a log‐rank test (*P* = .0396).

### 3.3 Associated factors of gastric cancer stage

We conducted a multivariate analysis of the 142 patients (89 in the persistent infection group and 53 in the successful eradication therapy group) shown in Table 2 to confirm the associated factors of stage I cancer after adjustment for age, sex, BMI, smoking habits, alcohol consumption, endoscopy within 2 years before cancer diagnosis, presence of open type atrophic gastritis, and successful *H. pylori* eradication therapy history before the gastric cancer diagnosis. As shown in Table 3, endoscopy within 2 years is associated with stage I. The other factors did not statistically affect the cancer stage.

**Table 3:** Multivariate analysis for associated factors of cancer stage I

Total (n = 142)	Multivariate	*P*
	OR (95% CI)	
Age (y)	1.01 (0.96‐1.05)	.6434
Male sex	0.86 (0.25‐2.87)	.8077
BMI (kg/m^2^)	1.15 (0.93‐1.43)	.1739
Smoker	0.84 (0.26‐2.67)	.7741
Alcohol consumer	1.53 (0.52‐4.53)	.4345
Endoscopy within 2 y	12.2 (1.25‐119.9)	.0314^a^
Atrophic gastritis, open type	0.87 (0.16‐4.47)	.8677
Successful *Helicobacter pylori* eradication therapy	1.51 (0.31‐7.34)	.6059

BMI, body mass index.

a Statistically significant.

## 4 DISCUSSION

We examined the prognosis of gastric cancer patients diagnosed after successful *H. pylori* eradication therapy and found that these gastric cancer patients showed better prognosis than the gastric cancer patients who did not undergo successful eradication therapy.

In this study, 86.8% of gastric cancers diagnosed after successful eradication therapy were the intestinal type (Table 2), and it was more frequent than the diffuse type. This is consistent with past reports.^10^, ^13^, ^23^

Our study revealed that gastric cancer patients diagnosed after successful eradication therapy showed early stage cancer at diagnosis and high a proportion of them received curative endoscopic therapy (Table 2). Decreased Prostate Stem Cell Antigen (PSCA) expression has been documented in gastric cancer^24^ and we recently showed that *H. pylori* eradication therapy resulted in increased PSCA expression.^25^

The other molecular biological effects of *H. pylori* eradication treatment on stomach cancer carcinogenesis and progression, which could not be evaluated in this research, have been vigorously investigated.^26^, ^27^, ^28^ Researchers have also reported that after *H. pylori* eradication, gastric atrophy decreases gradually and significantly.^29^, ^30^ Although the inhibitory effect on the development of gastric cancer following *H. pylori* eradication, which alters the biological characteristics and surrounding environment of the gastric cancer (as described above), is expected to improve prognosis, the present study failed to show this. Instead, we showed that patients who received successful *H. pylori* eradication therapy tended to undergo endoscopy in the 2 years prior to gastric cancer diagnosis (Table 2) and “endoscopy within 2 years before cancer diagnosis” contributed to early diagnosis of gastric cancer (Table 3). Representative guidelines recommend *H. pylori* eradication to reduce the gastric cancer incidence, and regular endoscopic surveillance for early detection and early treatment.^31^, ^32^ We believe our results support the important role of endoscopic surveillance after eradication therapy.

There were limitations to our study. First, the study participants were from a single outpatient endoscopic clinic. Future large‐scale research is needed. Second, we could not distinguish whether the gastric cancers diagnosed after eradication had occurred after eradication or existed before the eradication. Third, patients did not randomly receive *H. pylori* eradication therapy, and thus, there were background differences in the patients who received therapy and those who did not, such as the interval between endoscopic examinations. We are planning to conduct a further analysis that involves a greater number of cases and matches the backgrounds of the eradication group and natural history group as closely as possible.

## 5 CONCLUSION

In conclusion, we found that patients with gastric cancer, diagnosed after successful *H. pylori* eradication therapy, had a low mortality rate. Although the mortality rate was significantly decreased in the successful *H. pylori* eradication therapy group, the “endoscopy within 2 years before cancer diagnosis” was a confounding factor. That periodic endoscopy was associated with eradication treatment suggest that it may also lead to a reduction in the mortality rate and is therefore recommended.

## DISCLOSURES OF INTERESTS

The authors have no competing interests.
