# Supplementary material for: Early detection of gastric cancer after Helicobacter pylori eradication due to endoscopic surveillance
Source: Helicobacter. 2018 Jun 20;23(4):e12503. doi: 10.1111/hel.12503 (PMC6055630; doi:10.1111/hel.12503)
Supplement: Supplementary file 1 [file HEL-23-na-s001.docx]

Supplemental Table 1. Characteristics of the 11 deaths after gastric cancer diagnosis

|  | Age | Sex | Cause of death | Stage | Treatment | Observe (m) |
| --- | --- | --- | --- | --- | --- | --- |
| 1 | 43 | F | Gastric cancer | IV | Chemotherapy | 18.5 |
| 2 | 49 | F | Gastric cancer | IV | Chemotherapy | 18.2 |
| 3 | 65 | F | Gastric cancer | IV | Chemotherapy | 29.0 |
| 4 | 72 | M | Gastric cancer | IV | Chemotherapy | 6.3 |
| 5 | 70 | F | Gastric cancer | IV | Best supportive care | 4.6 |
| 6 | 87 | F | Gastric cancer | IV | Best supportive care | 1.8 |
| 7 | 70 | F | Gastric cancer | III | Observation | 25.6 |
| 8 | 79 | M | Pneumonia | III | Surgery | 14.9 |
| 9 | 84 | M | Myocardial infarction | I | Observation | 16.1 |
| 10 | 77 | M | Pharyngeal cancer | I | Endoscopy | 34.1 |
| 11 | 82 | F | Senility | I | Endoscopy | 44.3 |
